# Associations between industry involvement and study characteristics at the time of trial registration in biomedical research

**DOI:** 10.1371/journal.pone.0222117

**Published:** 2019-09-25

**Authors:** Anna Lene Seidler, Kylie E. Hunter, Nicholas Chartres, Lisa M. Askie

**Affiliations:** 1 NHMRC Clinical Trials Centre, University of Sydney, Sydney, Australia; 2 The University of Sydney, Sydney, Australia; University of Oxford, UNITED KINGDOM

## Abstract

**Background:**

Commercial or industry funding is associated with outcomes that favour the study funder in published studies, across various areas of research. However, it is currently unclear whether there are differences between trials with and without industry involvement at the stage of trial registration.

**Objective:**

To determine whether industry involvement (industry sponsorship, funding, or collaboration) is associated with trial characteristics at the time of trial registration.

**Methods:**

We conducted a cross-sectional analysis of all interventional studies registered on the Australian New Zealand Clinical Trials Registry in 2017 and classified them by industry involvement. We analysed whether there were differences in study characteristics (including type of control, sample size, study phase, randomisation, registration timing, and purpose of study) by industry involvement.

**Results:**

Industry involvement was reported by 21% of the 1,433 included trials. Only 40% of trials with industry involvement used an active control compared to 58% of non-industry trials (OR = 0.49, 95%CI = 0.38 to 0.63, p < .001), and industry trials reported smaller sample sizes (Median(IQR)_industry_ = 45(24–100), Median(IQR)_non-industry_ = 70(35–160), Mean Difference = -153, 95% CI = -233 to -75, p < .001). Industry trials were more likely to be earlier phase trials (Χ^2^(df) = 71.46(4), p < .001). There was no difference in use of randomisation between industry (70%) and non-industry trials (73%) (OR = 0.88, 95%CI = 0.67–1.20, p = .38). Eighty-three percent of industry trials compared to 70% of non-industry trials were prospectively registered (OR = 2.02, 95%CI = 1.47–2.82, p < .001). Industry trials were more likely to assess treatment (85%), rather than prevention, education or diagnosis compared to non-industry trials (64%) (OR = 3.02, 95%CI = 2.17–4.32, p < .001).

**Conclusion:**

The current study gives insight into differences in trial characteristics by industry involvement at registration stage. There was a reduced use of active controls in trials with industry involvement which has previously been proposed as a mechanism behind more favourable results. Non-industry funders and sponsors are crucial to ensure research addresses not only treatments, but also prevention, diagnosis and education questions.

## Introduction

A large proportion of clinical trials are funded by the commercial sector. For instance, in the United States 70% of money for drug trials is provided by industry.[1] The impact of industry involvement on study outcomes and how best to manage this has been widely debated in the scientific community.[2, 3] Some see industry involvement as necessary so that researchers and industry funders can fulfil their joint mission of fighting human disease. [4] Others are concerned that the strong financial incentive of industry-funded trials may threaten the credibility of research and thus poses a risk to evidence-based medicine.[5]

Previous empirical examinations of industry- and non-industry-funded pharmaceutical, tobacco and chemical research found that industry funding was associated with outcomes that favoured the commercial funder, even when controlling for other biases in the methods.[6–14] A Cochrane review examining the association of industry funding and favourable outcomes in primary studies of drug or medical devices across different fields of research found that industry-funded studies were more likely to report favourable efficacy results (risk ratio (RR) = 1.27, confidence interval (CI) = 1.21 to 1.44).[15] These differences between industry and non-industry funded research could not be explained by methodological biases. Studies using the Cochrane risk of bias tool found no difference on the domains of allocation sequence concealment, sequence generation, or loss to follow-up, and industry funded trials were of lower risk of bias in the blinding domains.[15]

This effect has previously been named ‘funding bias’ and is evident not only when comparing industry funded to non-industry funded trials, but also when comparing trials that were funded by different commercial companies.[2] For instance, in head-to-head comparisons of industry funded trials examining statins, results were more likely to favour the funder’s drug compared to the competitor drug.[16] Mechanisms beyond the traditional risk of bias tool have been proposed that may explain this funding bias, including systematic differences in study design, conduct, and the reporting of results.[17] These mechanisms include the choice of an inappropriate control, conducting many small trials to then selectively publish the ones that yield impressive results, and putting a spin on conclusions.[5, 17, 18]

Clinical trial registries are a valuable resource for exploring the landscape of clinical trials. The International Committee of Medical Journal Editors (ICMJE) requirement of prospective trial registration [19] and the recognition of clinical trial registration as an ethical requirement [20, 21] have led to an increase in registration rates over the last decade. A recent study audited registration status of all clinical trials published in 28 general and specialty, high- and low impact journals from January to June 2017. Of the audited trials, 95% of trials were registered on a World Health Organisation recognized clinical trials registry. To date, there has been one study using trial registry data (from the US registry ClinicalTrials.gov) to examine characteristics of drug trials depending on industry funding.[22] This study was however restricted to five drug categories; and it included only trials registered up to 2006, a time at which trial registration was not yet generally required and thus registration rates were low.

The Australian New Zealand Clinical Trials Registry (ANZCTR) routinely collects detailed information on the involvement of the commercial sector in trials, as well as the following trial characteristics relevant to funding bias: *(1) Type of control and sample size* are mechanisms that have previously been proposed to explain funding bias.[5, 17] ANZCTR data allow to systematically assess whether type of control and sample size differ depending on industry involvement. (2) *Randomised allocation and registration timing* are two characteristics assessed on the Cochrane risk of bias tool^,23^ (in the domains allocation concealment and selective reporting). For published trials, funding bias is not evident in the traditional risk of bias assessment domains of the Cochrane risk of bias tool,[15, 23] but to date it is unclear whether there are differences at registration stage. (3) *Study phase and purpose* can indicate whether the general aim and type of trial differ for trials conducted by industry as opposed to trials conducted by non-industry stakeholders such as universities or governments.

The aim of the current study was to determine whether industry involvement (industry sponsorship, funding, or collaboration) is associated with trial characteristics relevant to funding bias (type of control, sample size, study phase, randomised allocation, registration timing and study purpose).

## Methods

### Study design, eligibility criteria and data source

This was a cross-sectional analysis, including all interventional studies that were registered on the ANZCTR in 2017 (this includes published and unpublished studies). Observational studies were excluded, since many of the examined study characteristics (e.g. type of control, randomisation) do not apply to observational studies. All measures were extracted directly from the ANZCTR database (which contains raw, row-by-row data for all ANZCTR registry records) into a comma-separated values (csv) data file.

The ANZCTR is a Primary Registry in the World Health Organisation Registry Network. It accepts trial registrations from all over the world, but over 80% of all trials registered on the ANZCTR are Australian or New Zealand trials.[24]

### Measures

#### Classification of industry involvement

The ANZCTR collects information on funding, sponsorship and collaborators:

- *Funding* is defined as financial/ material or infrastructure support, and each study can list multiple funding sources.
- *Primary sponsor* is the individual or organisation initiating and managing the study, usually the principal investigator. Only one primary sponsor can be selected on the ANZCTR. Whilst this term has been used differently across the literature, on the ANZCTR the primary sponsor carries the main responsibility but does not necessarily fund the study.
- *Secondary sponsor* is defined as additional individuals or organisations that have agreed with the primary sponsor to jointly take on responsibilities of sponsorship. Studies can list one, none, or multiple secondary sponsors.
- *Collaborators* are individuals or organisations that have also agreed to take on responsibilities of sponsorship. Multiple entries are possible for this field.

For each of these fields, registrants select one of the following options: Commercial sector/industry, University, Government body, Hospital, Individual (which may for instance be an academic lead acting as sponsor for a trial involving multiple stakeholders), Charities/societies/foundations, Other collaborative groups, Other. For the purposes of this study, ‘Other collaborative groups’ and ‘Other’ have been merged to a single field ‘Other’.

Registrants also give further detailed information (name and contact information) for each involved stakeholder in a free-text field. Information is quality-checked and if necessary queried by ANZCTR staff before being approved for registration.

For this study, a new measure *any industry involvement* was computed, indicating whether ‘Commercial sector/ industry’ was listed in any of the above fields (i.e. whether there was any industry funding, sponsorship, or collaboration).

#### Study characteristics

The following study characteristics measures were included in the analysis:

*Type of control* is the type of treatment against which the intervention is being compared, categorised as: *Placebo* (inactive or sham treatment), *Active* (such as standard care, alternate form of treatment, dose comparison), *Uncontrolled* (same intervention applied to all subjects), *No treatment* (the control group received no treatment), and *Other* (such as historical control groups).

*Target sample size* was defined as the anticipated number of participants per trial. This was included rather than actual sample size given many of the trials had not completed recruitment at time of analysis since they were registered in 2017. An additional variable was created indicating whether target sample size was above or below the median of all studies.

*Study phase* was defined as the step at which research is conducted in treatment development. Phase 1 trials evaluate metabolism and pharmacological action of drugs, and monitor side effects. Phase 2 trials evaluate the effectiveness of new drugs in patients with the disease or condition being studied and to determine common short-term side effects and risks. Phase 3 involves the acquisition of additional information on benefits and risk, including possible adverse reactions. In Phase 4 trials, additional information is acquired after a drug has been marketed, monitoring aspects such as toxicity, risks, utility, benefits and optimal use. On the ANZCTR, ‘Phase’ is an optional field, and registrants can also choose combined phases (e.g. Phase 2/3). For this study, combined phases were re-grouped into the lower phase (e.g. all Phase 2/3 trials were categorised as Phase 2 trials).

*Randomised allocation* was defined as whether subjects were allocated randomly to their treatment group. In a *randomised-controlled trial*, subjects are allocated randomly to either the intervention or control group. In a *non-randomised trial* subjects are allocated deliberately, or not at random. This includes single-arm trials with no control group.

*Registration timing* was defined as whether the trial was registered *prospectively* (before enrolment of the first participant) or *retrospectively* (after enrolment of the first participant).

*Study purpose* includes the categories *treatment (*studies designed to evaluate interventions for treating a health condition), *prevention (*studies designed to assess interventions aimed at preventing the development of a disease or health condition), *diagnosis (*studies designed to evaluate interventions aimed at identifying a disease or health condition), or *education/counselling/training (*studies designed to assess interventions in an educational, counselling or training environment).

### Analysis

The frequency and proportion of study characteristics was compared by industry involvement and also by primary sponsor type. For binary characteristics, we calculated odds ratios (OR) and 95% confidence intervals (CI) using a logistic regression model to measure the association between industry involvement or primary sponsor and trial characteristics. For analyses by primary sponsor, the largest group (university sponsor) was used as a reference group for logistic regression. For categorical outcomes, chi-square tests were performed to measure the association. For continuous measures, mean differences and 95% CI were calculated using linear regression models. A sensitivity analysis was conducted calculating the associations between trial characteristics and any industry funding (i.e. any financial/material/infrastructure support for the study from the commercial sector/ industry) instead of any industry involvement (as a sponsor, collaborator and/or funder). All analyses were conducted using the open-source software R.[25]

## Results

### Characteristics of included studies

We included a total of 1,433 interventional studies in our analyses. Of these, 300 (21%) reported any industry involvement (industry funding, sponsorship and/or collaborator). Of the trials with industry involvement, the majority (n = 285, 95%) reported industry funding, about half (n = 153, 51%) reported a primary industry sponsor, and fewer reported a secondary industry sponsor (n = 50, 17%) or an industry collaborator (n = 12, 4%). For primary sponsor type, university sponsorship was reported most commonly (n = 541, 38%), followed by individual sponsors (n = 318, 22%), hospitals (n = 253, 18%), commercial sector/industry (n = 153, 11%) government bodies (n = 53, 4%), and charities/societies/foundations (n = 24, 2%). The remaining 91 trials (6%) listed ‘other’ as their primary sponsor.

### Trial characteristics by industry involvement and primary sponsor type

**Fig 1:**
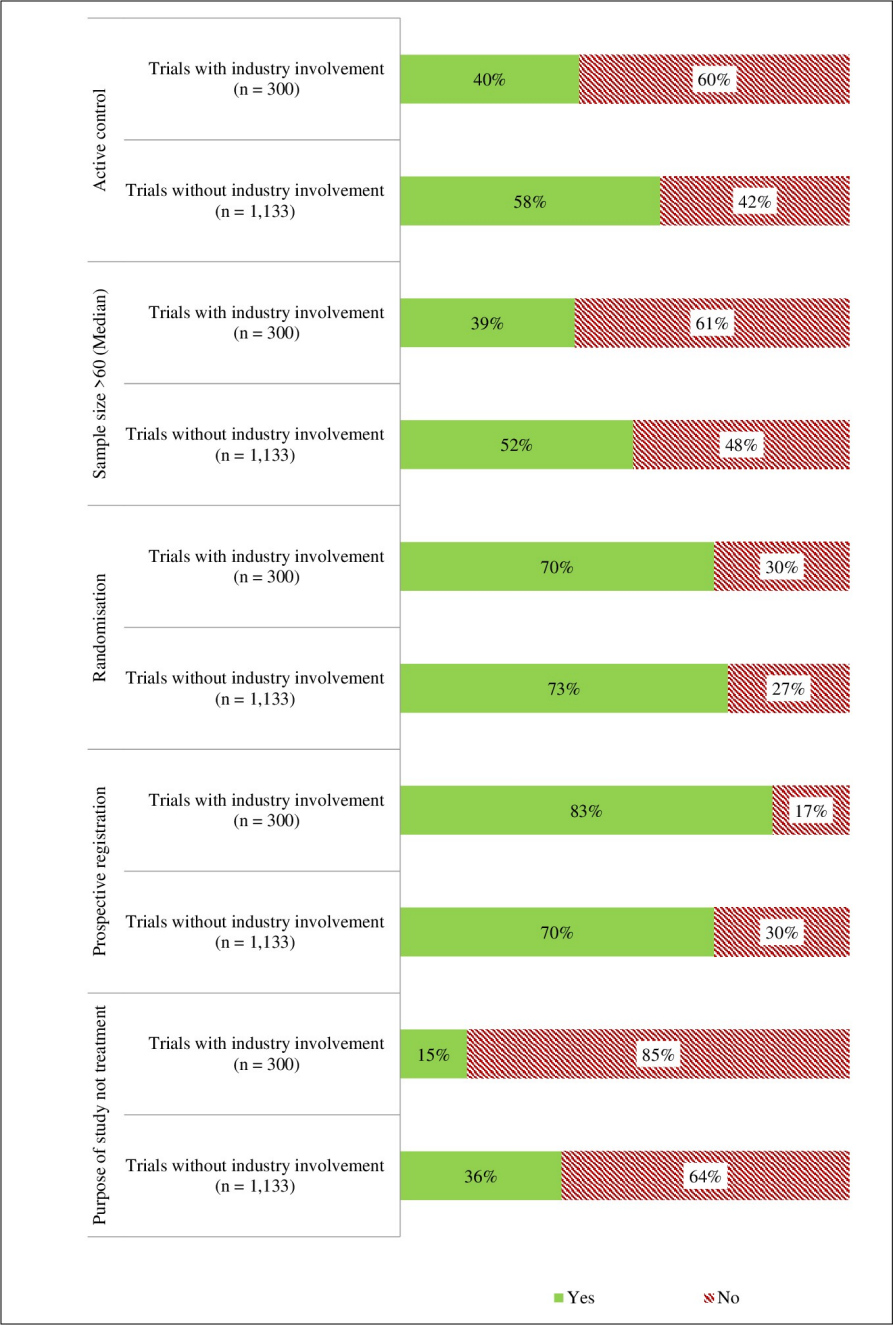
Trial characteristics by industry involvement.

Frequencies for trial characteristics by industry involvement are shown in Fig 1 and Table A in S1 File , and frequencies for trial characteristics by primary sponsor type are shown in Table B in S1 File

#### Type of control by industry involvement and primary sponsor type

**Fig 2:**
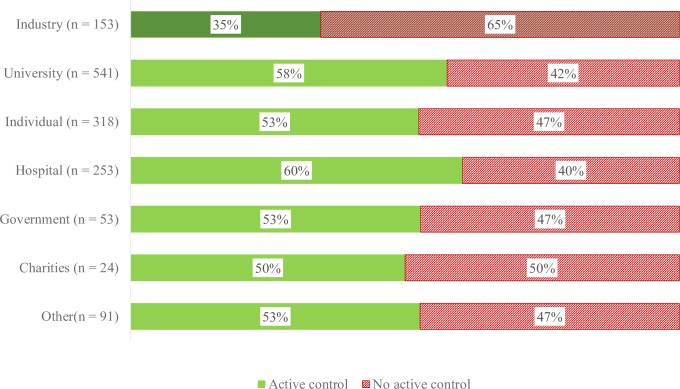
Control group by primary sponsor type.

Trials with industry involvement were less likely to use active controls (40%) compared to trials without industry involvement (58%) (OR = 0.49, 95% CI = 0.38 to 0.63, p < .001, Fig 1), and trials that reported an industry primary sponsor (35%) were less likely to use an active control than trials that reported a non-industry primary sponsor (56%) (OR = 0.39, 95% CI = 0.27 to 0.56, p < .001, Fig 2).

#### Target sample size by industry involvement and primary sponsor type

Trials with industry involvement were smaller on average. They had a median sample size of 45 (Interquartile range [IQR] = 24–100) whilst trials without industry involvement had a median sample size of 70 (IQR = 35–160). The mean difference between trials with and without industry involvement was -153 (95% CI = -231 to -74, p < .001). Trials with an industry primary sponsor were less likely to have a sample size above the median of 60 (OR = 0.28, 95% CI = 0.19 to 0.42, p < .001).

#### Study phase by industry involvement and primary sponsor type

**Fig 3:**
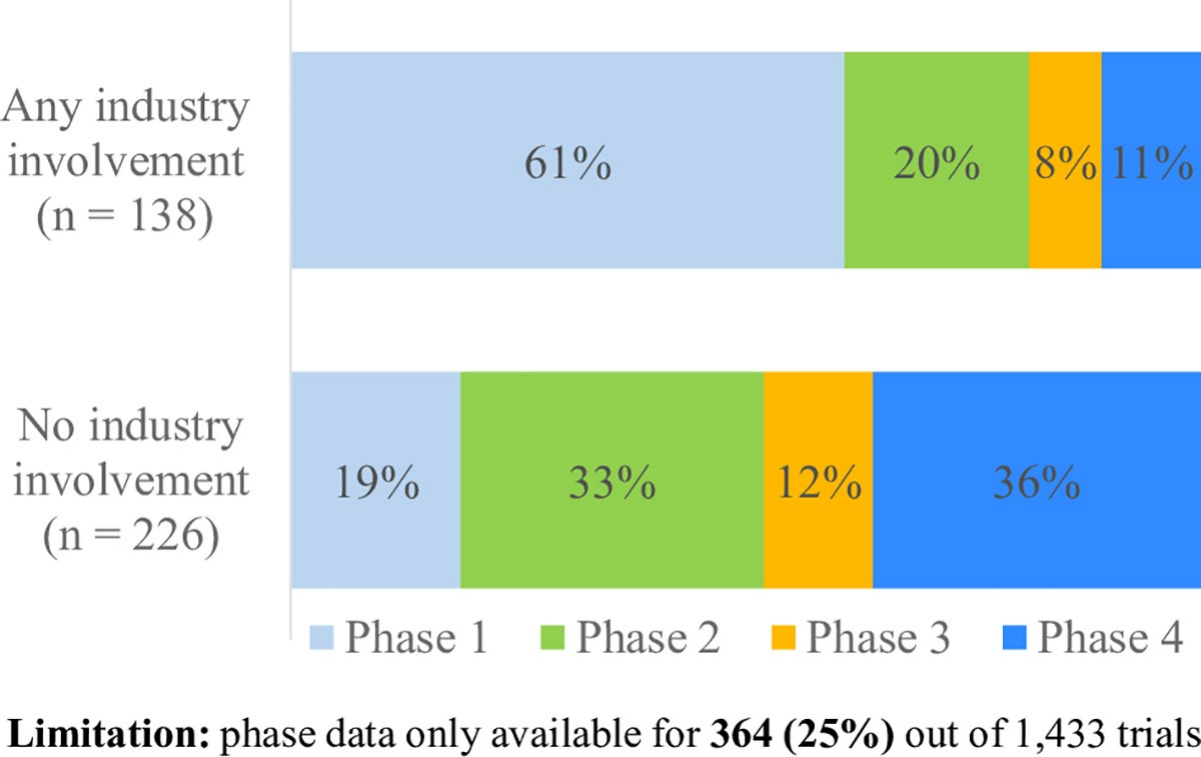
Trial phases by industry involvement.

Since trial phase is an optional field on the ANZCTR, and does not apply to non-drug trials, information on trial phase was only available for 364 (25%) of the included trials. For trials with data available, industry involvement was significantly associated with study phase (Fig 3, Χ^2^ (df) = 71.46(4), p < .001). Trials with industry involvement were more likely to be early trials (Phase 1) (61% of trials with available phase data), whilst trials without industry involvement were more likely to be post-marketing trials (Phase 4) (36%).

#### Randomised allocation by industry involvement and primary sponsor type

There was no significant difference between trials with (70%) and without industry involvement (73%) for randomised allocation (OR = 0.88, 95% CI 0.67 to 1.20, p = .38). Similarly, there was no difference by primary sponsor type with 72% of trials with an industry primary sponsor and 72% of trials without an industry primary sponsor using randomisation (OR = 0.73, 95% CI = 0.49 to 1.10, p = .13).

#### Registration timing by industry involvement and primary sponsor type

Trials with any industry involvement were more likely to be prospectively registered (83%) when compared to those with no industry involvement (70%) (OR = 2.02, 95% CI 1.47 to 2.82, p < .001). A similar association was found for trials that reported a primary industry sponsor (84% prospectively registered) compared to trials with a university as their primary sponsor (72%) (OR = 1.96, 95% CI = 1.25 to 3.20, p = .005).

#### Study purpose by industry involvement and primary sponsor type

**Fig 4:**
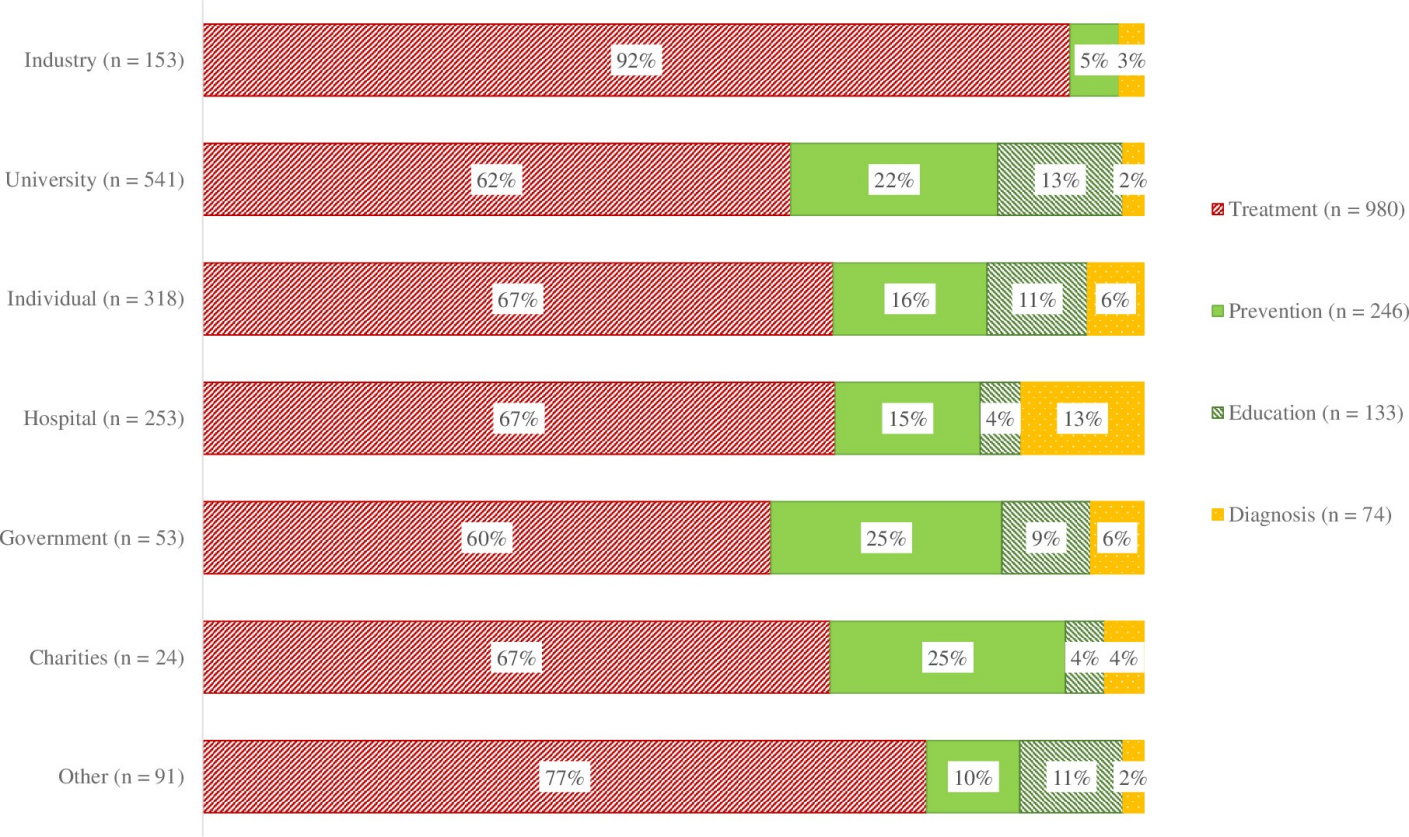
Study purpose by primary sponsor type.

As shown in Fig 4, trials with industry involvement were more likely to be aimed at treatment (83%) and less likely to assess prevention, education/counselling/training or diagnosis as their purpose compared to non-industry trials of which 70% were aimed at treatment (OR = 2.68, 95% CI = 1.95 to 3.75, p < .001). Similarly, trials with an industry primary sponsor were more likely to be aimed at treatment with 92% of trials with an industry primary sponsor aimed at treatment compared to 62% of trials with a university as the primary sponsor (62%) (OR = 7.06, 95% CI = 3.97 to 13.72, p < .001).

### Sensitivity analysis

We performed a sensitivity analysis examining associations between any industry funding (instead of any industry involvement) with trial characteristics. This did not change any of the proportions by more than two percentage points. This was to be expected since 95% of studies with industry involvement also reported industry funding.

## Discussion

In 2017, 21% of all interventional studies registered on the ANZCTR reported industry involvement, and for 11% the individual or organisation taking primary responsibility for the study (i.e. the primary sponsor) was from the commercial sector/ industry. Industry trials differed from non-industry trials for a range of trial characteristics. Trials with industry involvement were smaller on average and less likely to use an active comparator, and they were more likely to be early phase trials and to be prospectively registered. Trials with industry involvement were more likely to be aimed at treatment, and less likely to list prevention, education/counselling/training or diagnosis as their primary purpose. These differences were even more pronounced when comparing trials with an industry primary sponsor to trials with a non-industry primary sponsor.

### Strengths and weaknesses

This study used a complete dataset of 1,433 interventional trials registered on the ANZCTR in 2017. All data were quality-checked and if necessary queried by ANZCTR staff prior to being approved for registration. Thus, data quality was high, and there were little to no missing values for most variables, apart from missing values for fields that were non-applicable to some of the studies (e.g. study phase only applied to drug trials). The dataset contained a range of key metrics to assess different types of industry involvement and various trial characteristics of interest. Examining interventional trials at registration allowed a unique insight into associations between industry involvement and trial characteristics at an early stage, often before trial results were known and thus before studies could have been selectively reported based on their results (i.e. publication or selective reporting bias).

There were also some limitations to this study. This was a cross-sectional study reporting unadjusted associations between funding source and trial characteristics. The results are useful for descriptive purposes, however, they should not be interpreted causally. For instance, industry funded trials were more likely to be earlier phase trials but also had a smaller sample size. For earlier phase trials smaller sample sizes may be more appropriate, and thus, the smaller sample sizes may be a result of a larger number of earlier phase trials. For drug trials, we were unable to differentiate between trials for new active substances on the one hand, and new indications for drugs that were already on the market on the other hand. If the commercial sector were more likely to support and conduct drug trials for one of these purposes that may affect trial characteristics.

The ANZCTR is one of 16 WHO Primary Registries, and 80% of studies registered on the ANZCTR are Australian or New Zealand trials. It is possible, that studies registered on the ANZCTR are different to studies registered on other registries. For instance, a previous study found the proportion of industry-funded trials on the US-registry, ClinicalTrials.gov, to be 44% [26] which is higher than the rate of 21% that we observed on the ANZCTR in this study. Future studies may thus examine the association between industry involvement and trial characteristics in other registries.

This study only includes registered clinical trials. Whilst clinical trial registration is an ethical requirement, not all trials comply with this requirement to date. Trials that were not registered could not be included in this study. Yet, a recent audit of 28 high- and low-impact factor general and specialty medicine journals found that registration rates were high—over 95% of published trials were registered on a WHO recognized registry.[27]

### Interpretation and implications

This study found an association between industry involvement and lower use of active controls. The use of non-active controls has previously been suggested as a potential mechanism that may partly explain funding bias: comparing a new treatment to a placebo as opposed to the current gold standard treatment (which would usually be some kind of active control) is likely to yield larger effect sizes and has higher chances of reaching statistical significance. [5, 17] Another potential mechanism that has previously been suggested is the conduct of multiple small trials and selectively publishing the ones that yield favourable results. Again, we found an association between industry involvement and smaller sample sizes. Yet, it is important to note that we are presenting descriptive associations in this study and thus these results need to be interpreted with caution. For some conditions there is no current gold standard treatment and thus a placebo control is the best available comparator. Similarly and as discussed above, the association between industry involvement and sample sizes may be explained by industry being involved in earlier phase trials, or it may be that later phase industry trials are more likely to be registered on other registries. Nonetheless, it may be appropriate to pay particular attention to the appropriate use of controls and sufficient sample size when assessing the methodological quality of trials with industry involvement on a case-by-case basis.

Previous studies have reported that funding bias was not evident in traditional risk of bias assessment domains such as the Cochrane risk of bias tool.[23] This study examined two variables that would be assessed in the Cochrane risk of bias tool: randomisation (Cochrane risk of bias tool: allocation concealment) and prospective registration (Cochrane risk of bias tool: selective reporting). We found no association between industry involvement and randomised allocation and trials with industry involvement were more likely to be prospectively registered. This confirms the previous finding that funding bias does not appear to be reflected on ‘traditional’ risk of bias assessment tools.

There was a strong association between industry involvement and the primary purpose of the study. Only 17% of trials with industry involvement (compared with 35% of trials without industry involvement) reported an aim other than treatment. This was even more pronounced when examining studies that had an industry primary sponsor, of which only 8% reported an aim other than treatment. The commercial sector needs to invest in clinical trials that are promising to be financially lucrative, and these may most often be related to treatment. Yet, prevention and education are crucial for population health and lead to lower demands and costs for public healthcare systems. Non-industry research is therefore important to ensure that research does not only address treatment, but also prevention and education questions.

### Conclusion

The current study gives insight to differences in trial characteristics by industry involvement at design stage. There was a reduced use of active controls in trials with industry involvement which has previously been proposed as a mechanism behind more favourable results. Non-industry funders and sponsors are needed to ensure research addresses not only treatment, but also prevention and education questions.

## Supporting information

S1 File Table A. Study characteristics by industry involvement and Table B. Study characteristics by primary sponsor type.
(DOCX)

S2 File Raw data. (XLSX)
