# Supplementary material for: Associations between industry involvement and study characteristics at the time of trial registration in biomedical research
Source: PLoS One. 2019 Sep 25;14(9):e0222117. doi: 10.1371/journal.pone.0222117 (PMC6760823; doi:10.1371/journal.pone.0222117)
Supplement: S1 File — Table A. Study characteristics by industry involvement and Table B. Study characteristics by primary sponsor type. (DOCX) [file pone.0222117.s001.docx]

**Table A.** **Study characteristics by industry involvement.**

|  | Any industry involvement  (n = 300) | No industry involvement  (n = 1,133) |
| --- | --- | --- |
| Type of control |  |  |
| Active (n = 773), *n(%)* | 120 (40%) | 653 (58%) |
| Placebo (n = 251), *n(%)* | 99 (33%) | 152 (13%) |
| No treatment (n = 127), *n(%)* | 10 (3%) | 117 (10%) |
| Uncontrolled (n = 248), *n(%)* | 66 (22%) | 182 (16%) |
| Other (n = 34), *n(%)* | 4 (1%) | 24 (2%) |
| Missing (n = 6), *n(%)* | *1 (0.3%)* | *5 (0.4%)* |
| *Χ^2^(df), p-value* | *Χ^2^(4) = 84.23, p <.001* | |
| *OR, 95% CI: active vs non-active* | *OR = 0.49, 95% CI = 0.38 to 0.63* | |
| Study phase |  |  |
| Phase 1 (n = 126), *n(%)* | 84 (28%) | 42 (4%) |
| Phase 2 (n = 103), *n(%)* | 28 (9%) | 75 (7%) |
| Phase 3(n = 38), *n(%)* | 11 (4%) | 27 (2%) |
| Phase 4 (n = 96), *n(%)* | 15 (5%) | 81 (7%) |
| Non-applicable/ missing (n = 1,069), *n(%)* | 162 (54%) | 907 (80%) |
| *Χ^2^(df), p-value* | *Χ^2^(4) = 71.46, p <.001* | |
| Sample size |  |  |
| Median (IQR) | 45 (24-100) | 70 (35-160) |
| Mean (SD) | 96 (198) | 249 (689) |
| ≤60 (n = 721), *n(%)* | 182 (61%) | 539 (48%) |
| >60 (n = 712), *n(%)* | 118 (39%) | 594 (52%) |
| *OR, 95% CI: above or below 60 (Median)* | *OR = 0.59, 95% CI = 0.45 to 0.76* | |
| *MD (95% CI)* | *MD = -152.99, 95% CI = -231.99 to -74.98* | |
| Randomisation |  |  |
| Randomised-controlled (n = 1,037), *n(%)* | 211 (70%) | 826 (73%) |
| Non-randomised (n = 396), *n(%)* | 89 (30%) | 307 (27%) |
| *OR, 95% CI: randomised vs non-randomised* | *OR = 0.88, 95% CI = 0.67 to 1.20* | |
| Registration timing |  |  |
| Prospective (n = 1,044), *n(%)* | 248 (83%) | 796 (70%) |
| Retrospective (n = 389), *n(%)* | 52 (17%) | 337 (30%) |
| *OR, 95% CI: prospective vs retrospective* | *OR = 2.02, 95% CI = 1.47 to 2.82* | |
| Purpose of study |  |  |
| Treatment (n = 980), *n(%)* | 249 (83%) | 731 (65%) |
| Prevention (n = 246), *n(%)* | 34 (11%) | 212 (19%) |
| Education/counselling/training (n = 133), *n(%)* | 5 (2%) | 128 (11%) |
| Diagnosis (n = 74), *n(%)* | 12 (4%) | 62 (5%) |
| *Χ^2^(df), p-value* | *Χ^2^(3) = 44.07, p < .001* | |
| *OR, 95% CI: treatment vs other purpose* | *OR = 2.68, 95% CI = 1.96 to 3.75* | |

Note: Odds ratios and chi-square values were calculated omitting missing cases from the dataset. OR = Odds Ratio; CI = Confidence Intervals; df = degrees of freedom. For the mean and mean difference calculations extreme outliers with sample sizes of 10,000 and above (n = 6) were set as missing

**Table B.** **Study characteristics by primary sponsor type**

|  | University  (n = 541) | Commercial sector/ industry  (n = 153) | Government  (n = 53) | Hospital  (n = 253) | Charities/societies/ foundations  (n = 24) | Individual  (n = 318) | Other  (n = 91) |
| --- | --- | --- | --- | --- | --- | --- | --- |
| Type of control |  |  |  |  |  |  |  |
| Active (n = 773), *n(%)* | 312 (58%) | 53 (35%) | 28 (53%) | 153 (60%) | 12 (50%) | 167 (53%) | 48 (53%) |
| Placebo *(n = 251), n(%)* | 87 (16%) | 58 (38%) | 6 (11%) | 25 (10%) | 4 (17%) | 52 (16%) | 19 (21%) |
| No treatment (n = 127), *n(%)* | 66 (12%) | 3 (2%) | 3 (6%) | 15 (6%) | 1 (4%) | 36 (11%) | 3 (3%) |
| Uncontrolled (n = 248), *n(%)* | 65 (12%) | 38 (25%) | 16 (30%) | 54 (21%) | 7 (29%) | 49 (15%) | 19 (21%) |
| Other (n = 34), *n(%)* | 9 (2%) | 0 (0%) | 0 (0%) | 5 (2%) | 0 (0%) | 12 (4%) | 2 (2%) |
| Missing (n = 6), *n(%)* | 2 (0%) | 1 (1%) | 0 (0%) | 1 (0%) | 0 (0%) | 2 (1%) | 0 (0%) |
| OR, 95% CI: active vs non-active | *1 (Reference)* | *0.39 (0.27 to 0.56)* | *0.82 (0.47 to 1.45)* | *1.12 (0.83 to 1.52)* | *0.73 (0.32 to 1.68)* | *0.81 (0.61 to 1.07)* | *0.82 (0.52 to 1.28)* |
| Χ^2^(df), p-value | *Χ^2^(24) = 120.36, p < .001* | | | | | | |
| Study phase |  |  |  |  |  |  |  |
| Phase 1 (n = 126), *n(%)* | 12 (2%) | 76 (50%) | 1 (2%) | 15 (6%) | 5 (20%) | 14 (4%) | 3 (3%) |
| Phase 2 (n = 103), *n(%)* | 26 (5%) | 15 (10%) | 5 (9%) | 25 (10%) | 1 (4%) | 16 (5%) | 15 (16%) |
| Phase 3(n = 38), *n(%)* | 13 (2%) | 5 (3%) | 0 (0%) | 4 (2%) | 1 (4%) | 7 (2%) | 8 (9%) |
| Phase 4 (n = 96), *n(%)* | 22 (4%) | 2 (1%) | 12 (23%) | 25 (10%) | 3 (12%) | 22 (7%) | 10 (11%) |
| Non-applicable (n = 1,069), *n(%)* | 468 (87%) | 55 (36%) | 35 (66%) | 184 (73%) | 15 (60%) | 259 (81%) | 55 (60%) |
| Χ^2^(df), p-value | *Χ^2^(18) = 142.04, p < .001* | | | | | | |
| Sample size |  |  |  |  |  |  |  |
| ≤60 (n = 721), *n(%)* | 240 (44%) | 113 (74%) | 20 (38%) | 143 (57%) | 15 (63%) | 150 (47%) | 40 (44%) |
| >60 (n = 712), *n(%)* | 301 (56%) | 40 (26%) | 33 (62%) | 110 (43%) | 9 (38%) | 168 (53%) | 51 (56%) |
| OR, 95% CI: above or below 60 (Median) | *1 (Reference)* | *0.28 (0.19 to 0.42)* | *1.32 (0.74 to 2.39)* | *0.61 (0.45 to 0.83)* | *0.48 (0.20 to 1.09)* | *0.89 (0.68 to 1.18)* | *1.02 (0.65 to 1.60)* |
| Randomisation |  |  |  |  |  |  |  |
| Randomised-controlled (n = 1,037), *n(%)* | 421 (78%) | 110 (72%) | 34 (64%) | 158 (62%) | 12 (50%) | 234 (74%) | 68 (75%) |
| Non-randomised (n = 396), *n(%)* | 120 (22%) | 43 (28%) | 19 (36%) | 95 (38%) | 12 (50%) | 84 (26%) | 23 (25%) |
| OR, 95% CI: randomised vs non-randomised | *1 (Reference)* | *0.73 (0.49 to 1.10)* | *0.51 (0.28 to 0.94)* | *0.47 (0.34 to 0.66)* | *0.29 (0.12 to 0.66)* | *0.79 (0.58 to 1.10)* | *0.84 (0.51 to 1.43)* |
| Registration timing |  |  |  |  |  |  |  |
| Prospective (n = 1,044), *n(%)* | 391 (72%) | 128 (84%) | 42 (79%) | 173 (68%) | 15 (63%) | 225 (71%) | 70 (77%) |
| Retrospective (n = 389), *n(%)* | 150 (28%) | 25 (16%) | 11 (21%) | 80 (32%) | 9 (38%) | 93 (29%) | 21 (23%) |
| OR, 95% CI: prospective vs retrospective | *1 (Reference)* | *1.96 (1.25 to 3.20)* | *1.47 (0.76 to 3.06)* | *0.83 (0.60 to 1.15)* | *0.64 (0.28 to 1.55)* | *0.93 (0.68 to 1.26)* | *1.28 (0.77 to 2.20)* |
| Purpose of study |  |  |  |  |  |  |  |
| Treatment (n = 980), *n(%)* | 338 (62%) | 141 (92%) | 32 (60%) | 170 (67%) | 16 (67%) | 213 (67%) | 70 (77%) |
| Prevention (n = 246), *n(%)* | 119 (22%) | 8 (5%) | 13 (25%) | 39 (15%) | 6 (25%) | 52 (16%) | 9 (10%) |
| Education (n = 133), *n(%)* | 72 (13%) | 0 (0%) | 5 (9%) | 11 (4%) | 1 (4%) | 34 (11%) | 10 (11%) |
| Diagnosis (n = 74), *n(%)* | 12 (2%) | 4 (3%) | 3 (6%) | 33 (13%) | 1 (4%) | 19 (6%) | 2 (2%) |
| OR, 95% CI: treatment vs other purpose | *1 (Reference)* | *7.06 (3.97 to 13.72)* | *0.92 (0.52 to 1.65)* | *1.23 (0.90 to 1.69)* | *1.20 (0.52 to 3.01)* | *1.22 (0.91 to 1.63)* | *2.00 (1.21 to 3.43)* |
| Χ^2^(df), p-value | *Χ^2^(18) = 118.39, p < .001* | | | | | | |

Note: Odds ratios and chi-square values were calculated omitting missing cases from the dataset. OR = Odds Ratio; CI = Confidence Intervals; df = degrees of freedom.
